# Evaluation of an Optimized Photobiomodulation Protocol for Accelerated Tooth Movement

**DOI:** 10.7759/cureus.101755

**Published:** 2026-01-17

**Authors:** Anupama V Jain, Shrinivas M Basavraddi, Roopak Naik

**Affiliations:** 1 Orthodontics and Dentofacial Orthopedics, Shree Dharmastala Manjunathesawara College of Dental Sciences, Dharwad, IND

**Keywords:** accelerated tooth movement, digital cast measurements, enmass space closure, low level laser therapy (lllt), nickel-titanium, orthodontics, pbm protocol, photobiomodulation

## Abstract

Introduction

Photobiomodulation (PBM) has a stimulatory effect on periodontal ligament cells involved in bone remodelling and acts synergistically with the applied orthodontic force to accelerate tooth movement and reduce treatment time. This study aims to compare the mean monthly rate of en-masse maxillary extraction-space closure over five months between PBM plus standard orthodontic force and standard force alone.

Material and methods

A randomised, parallel arm, single-blinded pilot study was carried out with control and PBM groups. For all patients after extraction of first bicuspids, a retraction force of 300g per side was applied using a nickel-titanium (NiTi) closed coil spring. Additionally, in the PBM group, PBM irradiation was done at two buccal and two palatal points along the roots for all six maxillary anterior teeth with an 810 nm diode laser (continuous wave (CW), 200 mW, 0.3cm^2^, 8 sec per spot, four spots per tooth, 6.4J per tooth) on the first, third, seventh, and 14th day from appliance activation day. Thus, appliance activation was done one time per month and four PBMs were done in a month for five consecutive months. The amount of anterior segment retraction and extraction space closure was measured for every patient on the maxillary right and left side at monthly intervals using a digital caliper (Method I) and 3D software (Method II). The collected data were tabulated and statistically analysed using SPSS 25.0 for Windows (IBM Corp., Armonk, NY, USA) and an independent sample t-test was performed (p <0.05).

Results

Comparison of laser versus control first month space closure in mm showed first month (T0-T1) (right side, Method I) (Mean±SD;1.26±-0.105 vs 0.53±0.45); first month (T0-T1) (Right side, Method II) (Mean±SD;1.06±0.15 vs 0.59±0.107). The mean mm rate of en-masse maxillary space closure (on the right and left side) measured by both Method I and Method II across all five months was consistently higher in the laser group compared to the control. The difference in the monthly rate of space closure was statistically significant (p<0.05). Mean differences with 95% CI are reported. The PBM group showed 1.09mm per month extraction space closure compared to 0.7mm per month in the control group. In the patients who received PBM, the tooth movement was 40% faster than in the control group.

Conclusion

PBM added to orthodontic force was associated with a higher monthly rate of extraction space closure. Based on these preliminary results and analysis, the PBM protocol with the new PBM device and the methodology implemented in this randomized pilot study can be replicated for larger trials to check effectiveness in accelerating tooth movement and reducing treatment time.

## Introduction

In patients complaining of proclination and forwardly placed front teeth, the routine treatment protocol is to extract the maxillary right and left first bicuspids and push the front teeth backwards using a fixed orthodontic appliance. The appliance used is braces and wires, which are fixed on the patient’s teeth along with springs and elastics that apply the pull force on the front teeth using molars as anchor teeth. The springs and elastics that create the pull force are stretched and activated once a month to maintain required force levels. The average orthodontic treatment duration is 24-36 months and due to this prolonged treatment duration, it has adverse effects [1]. These include difficulty in maintaining oral hygiene, increased caries incidence, white spot lesions, and root resorption, among others [2,3]. Hence, there is a need to reduce treatment time by accelerating the tooth movement.

Tooth movement is the result of the bone remodelling (bone deposition and resorption) induced by application of forces on the teeth which causes extracellular matrix deformation of the adjacent alveolar bone and phases of acute, then chronic inflammatory reaction involving the periodontal ligament cells (PDLCs; osteoblasts, osteocytes, fibroblasts) [4,5]. On the basis of the knowledge of underlying molecular pathways, various methods have been devised to accelerate the rate of tooth movement [6]. Invasive surgical methods like corticotomy, peizocision, periodontally accelerated osteogenic orthodontics (PAOO), micro-osteoperforations (MOP), and injectable platelet-rich fibrin (I-PRF) injections have drawbacks of unpleasant patient experience, pain, uncontrolled root resorption, etc. [7,8]. The non-invasive methods are low-level laser therapy (LLLT), high-frequency vibration devices, low-intensity pulsed ultrasound (LIPUS), pulsed magnetic field application, low amperage DC current and pharmacological methods [9]. Photobiomodulation (PBM), also called LLLT, is a safe non-invasive method when used along with pulling force of springs and elastics and has been found to increase the rate of tooth movement by stimulation of the bone remodelling cells without any adverse effects [10].

After the initial alignment and levelling of the dental arches, extraction space closure, where the forwardly placed front teeth are pulled (retracted) backwards, is the most prolonged phase of orthodontic treatment. Conventionally, it takes eight to 10 months on average to close the extraction spaces by pulling the front teeth backwards. Though many previous studies have shown lasers can accelerate tooth movement, only a few have explored their effect during the space closure stage. Also, most studies have focused on single tooth movement like canine retraction, a technique which is rarely used in regular orthodontic treatment because of the advent of absolute anchorage solutions like mini-implants and bone screws [11,12]. Previous studies also are of short duration of one to three months average [13-15]. A few studies on en-masse retraction using LLLT alone or a combination of techniques like LLLT and peizocision have been reported recently [16]. The varied laser irradiation protocols used in different studies have added to the uncertainty [17].

When the appliance fixed on the patient’s teeth is activated by stretching the springs and elastics, there is a retraction force applied on the front teeth. The ensuing tooth movement happens in phases - initial phase, lag phase, and post lag phase, and in each phase the cellular activity varies. The PBM protocol used in the study is based on the rationale of sequentially stimulating and activating at each phase all the different cells that bring about bone remodelling and tooth movement.

The primary objective of this study was to compare the mean monthly rate of maxillary extraction space closure over five months between PBM plus standard orthodontic force and standard force alone. Additionally, comparison of space closure measurements between the caliper measurement method (Method I) and the digital method (Method II) is done as a secondary outcome.

## Materials and methods

Patient enrollment, study design, sampling

**Figure 1:**
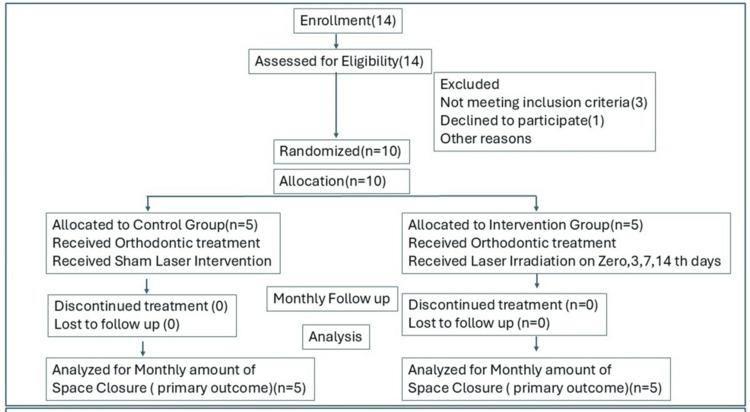
Consolidated Standards of Reporting Trials (CONSORT) flow diagram for randomised clinical feasibility trials. CONSORT guidelines 2010, updated 2025 [18]

The study was designed as single blinded (patient and outcome assessor blinded) two-arm parallel prospective pilot study following the Consolidated Standards of Reporting Trials (CONSORT) guidelines (Figure 1) [18].

Patients with maxillary anterior teeth proclination requiring orthodontic treatment and meeting the inclusion criteria were enrolled from those reporting to the Department of Orthodontics and Dentofacial Orthopaedics at Shree Dharmastala Manjunathesawara (SDM) College of Dental Cciences, SDM University, Dharwad, Karnataka, India. Institutional ethics committee clearance letter was received before recruiting patients for the study (approval SDMCDS IEC.No.2021/Dental/Ph.D/Orthodontics/01) and informed consent was obtained from all patients. Simple random sampling was used to ensure unbiased participant selection from the eligible patient pool, thereby improving the internal validity and reliability of the pilot randomized controlled trial (RCT) findings.

Inclusion criteria for eligibility assessment

Female patients from North Karnataka, India, who were visiting the hospital and in permanent dentition between age 17 and 25 years were screened and assessed for eligibility as per pre-defined inclusion criteria. All patients had bimaxillary protrusion with convex profile, protruding upper lips, and minimal crowding, which was assessed by irregularity index <3mm. Cephalometricaly all patients had SNA >850-870, SNB >820-840, ANB 20-40, and β angle 270-300, with proclined upper incisors (U1-NA angle 280-340) and lower incisors (L1-NB angle 300-350), and reduced interincisal angle 1220-1280°. All patients had Class I molar with increased overjet between 4-5mm and overbite 3-4mm. Patients had normal mandibular growth pattern indicated by Frankfurt mandibular plane angle (FMA) between 250-300 and Jarabak ratio 62-65%. All subjects included in the study had sound periodontal health indicated by absence of gingival inflammation (GI ≤ 1), probing pocket depth ≤ 3 mm, no clinical attachment loss, bleeding on probing < 10% of sites, and no radiographic evidence of alveolar bone loss. Patients with prior orthodontic treatment, craniofacial anomalies, significant skeletal discrepancies requiring orthognathic surgery, systemic disease affecting bone metabolism, or who were pregnant were excluded. Baseline values for the inclusion criteria and also the amount of space available at start of space closure on the right (T0 Right) and left (T0 Left) maxillary arch were compared between the control and laser group. Independent t-test to compare these baseline values showed no significant difference between control and laser groups, hence the baseline values were ensured to be similar at the start of space closure (Table 1).

**Table 1:** Comparison of inclusion criteria parameters baseline value independent t-test results showing no significant difference between control and laser group at baseline (p>0.05 is not significant). SNA, sella–nasion–point A angle; SNB, sella–nasion–point B angle; ANB, angle between points A and B relative to the sella–nasion plane; β angle, sagittal skeletal discrepancy angle formed by points A, B, and condylion; U1–NA, angle between the maxillary central incisor long axis and the nasion–point A line; L1–NB, angle between the mandibular central incisor long axis and the nasion–point B line; interincisal angle, angle between the long axes of the maxillary and mandibular central incisors; FMA, Frankfort–mandibular plane angle; T0 (Right), distance between cusp tip of upper right canine to mesiobuccal cusp of upper right first molar at start of retraction; T0 (Left), distance between cusp tip of upper left canine to mesiobuccal cusp of upper left first molar at start of retraction

Parameter	Threshold values	Group	N	Mean	Std. Deviation	p value	mean diff	95% CI of mean difference	
Little’s Irregularity Index		CONTROL	5	1.2	0.84	1	0	-0.978	0.978
		LASER	5	1.2	0.45	NS			
SNA		CONTROL	5	86.2	0.45	1	0	-0.978	0.978
		LASER	5	86.2	0.84	NS			
SNB		CONTROL	5	83.6	0.55	0.545	-0.2	-0.929	0.529
		LASER	5	83.8	0.45	NS			
ANB		CONTROL	5	3	1	0.74	0.2	-1.145	1.545
		LASER	5	2.8	0.84	NS			
β angle		CONTROL	5	28.8	1.3	0.792	-0.2	-1.895	1.495
		LASER	5	29	1	NS			
U1 to NA		CONTROL	5	33.2	0.84	0.724	-0.2	-1.463	1.063
		LASER	5	33.4	0.89	NS			
L1-NB angle		CONTROL	5	33.4	0.89	0.766	-0.2	-1.694	1.294
		LASER	5	33.6	1.14	NS			
Interincisal angle		CONTROL	5	126.2	1.64	0.803	-0.2	-1.986	1.586
		LASER	5	126.4	0.55	NS			
FMA		CONTROL	5	28	1.58	0.576	-0.6	-2.974	1.774
		LASER	5	28.6	1.67	NS			
Jarabak Ratio		CONTROL	5	63.8	0.45	0.373	-0.4	-1.378	0.578
		LASER	5	64.2	0.84	NS			
Overjet		CONTROL	5	4.2	0.45	0.545	-0.2	-0.929	0.529
		LASER	5	4.4	0.55	NS			
Overbite		CONTROL	5	3.6	0.55	0.545	-0.2	-0.929	0.529
		LASER	5	3.8	0.45	NS			
T0 (Right)		CONTROL	5	21.2	1.79	0.684	-0.4	-2.588	1.788
		LASER	5	21.6	1.14	NS			
T0 (Left)		CONTROL	5	21.2	1.48	0.108	-1.6	-3.637	0.437
		LASER	5	22.8	1.3	NS			

All participants were instructed to avoid hard and chewy foods (e.g., nuts, raw carrots, toffees) and to refrain from consuming non-vegetarian food items during the study period. This standard dietary advice minimized the influence of dietary factors on tooth movement. Instructions were provided verbally and reinforced at each monthly visit.

Sample size and power analysis

The effect size for the present study was derived from the randomized controlled clinical trial by AlSayed Hasan et al. [14], which evaluated the effectiveness of low-level laser therapy in accelerating orthodontic tooth movement. From the reported means and standard deviations of space closure rates between the laser and control groups, Cohen’s d was calculated using the formula* d=(M_1_-M_2_)/(SD_pooled_ )*; where M_1 _is the mean of the laser group and M_2_ is the mean of the control group; SD_pooled=√((SD_1_^2^+SD_2_^2^)/2).

This yielded an estimated effect size of approximately d = 1.90, indicating a large treatment effect. For the present pilot study, a slightly smaller and more conservative effect size of d = 1.77 was adopted to account for population variability and the exploratory nature of the research.

Sample size was estimated for a two-tailed independent t-test (α = 0.05) using the standard formula n_(per group)_=2×((Z_(1-α/2)_+Z_(1-β)_ )^2^)/d^2^.

Substituting the values for 80% power, n=2×((1.96+0.84)^2^)/((1.77)^2^)=2×((2.80)^2^)/3.13=2×2.50=5.0. Thus, five participants per group (total = 10) were required to achieve approximately 80% power to detect an effect size of 1.77 at α = 0.05.

To verify the adequacy of the chosen pilot sample size, we did a theoretical (a priori) power analysis using G*Power 3.1.9.7 (Heinrich-Heine-Universität Düsseldorf, Düsseldorf, Germany) for a two-tailed t-test (α = 0.05, d = 1.77, n = 5 per group) which showed that a total of 10 participants would yield an expected power of approximately 0.83 (83%) to detect a large effect, verifying the adequacy of the selected pilot sample size.

Randomisation and allocation

Ten recruited subjects (A-J) were randomly assigned to study/PBM and control groups (1:1) using computer-generated block randomization (blocks of 4 and 6) with allocation concealment by the sequentially numbered, opaque, sealed envelopes (SNOSE) method. The random sequence was generated by an independent statistician not involved in enrollment or assessment.

Orthodontic treatment

In both groups, after first premolar extraction, patients' teeth were bonded with 0.022 slot preadjusted edgewise appliance (Gemini, 3M Unitek, Maplewood, MN, USA). Appliance bonding and en-masse retraction were performed using mechanics like those mentioned by Khlef et al. [19]. The first molars on either side (upper and lower) were banded and transpalatal arch placed for anchorage control. The second molars were banded/bonded and tied to the first molars to reinforce anchorage. Levelling and alignment were performed using nickel-titanium (NiTi) and stainless steel archwires in the following sequence: 0.016-inch NiTi, 0.020-inch NiTi, 0.017 × 0.025-inch NiTi, 0.019 × 0.025-inch NiTi, and 0.017 × 0.025-inch stainless steel. Anterior teeth from canine to canine were ligated using figure-of-eight ligature ties. Subsequently, working archwire 0.019”x0.025” stainless steel wires were inserted with hooks crimped on the archwire between the lateral incisor and canine. The working archwire was left passive in situ for three weeks before initiating retraction. NiTi closed coil springs (Ormco, Brea, CA, USA) were engaged on the right and left sides of both maxillary and lower arches to deliver 300g of retraction force on the anterior teeth, 150g per side [20]. The NiTi springs were engaged between the archwire hooks and molar tube hooks on the right and left sides from canine to molar in the patient's mouth. Retraction force was measured using a Dontrix gauge. Patients were recalled at 30-day intervals for monthly appliance activation. At each activation the retraction force levels were checked and, if required, the NiTi springs were changed and reactivated to maintain the constant retraction force. If any breakage occurred, patients were asked to report to hospital immediately and the same was fixed. Reminders were given for all patients for the follow-up appointments and there were no missed appointments.

Photobiomodulation treatment (laser intervention)

**Figure 2:**
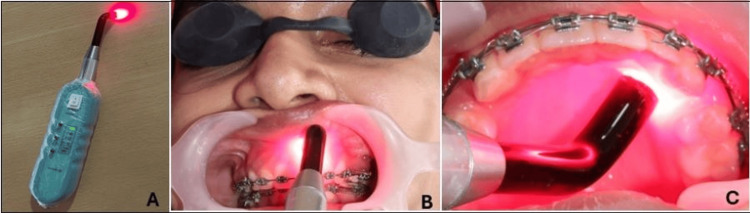
Photobiomodulation (PBM) treatment. A. PBM device N-lase (Medsol, Hosur, India). B. Low-level laser therapy (LLLT) irradiation along with anterior retraction using nickel-titanium (NiTi) coil springs (buccal side). C. LLLT irradiation along with anterior retraction using NiTi coil springs (palatal side). Protective eye wear being worn.

**Figure 3:**
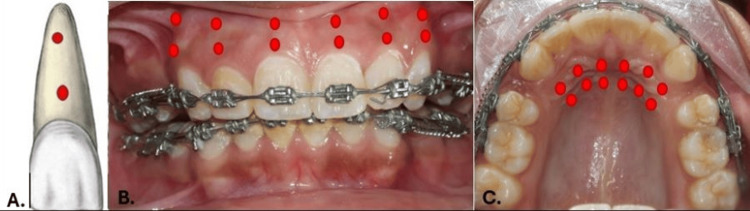
Low-level laser therapy (LLLT) irradiation method (schematic representation). A. Centre of cervical half and centre of apical half of each anterior tooth irradiated. B. Total of 12 buccal irradiation points. C. Total of 12 palatal irradiation points. Figure from [9]

The two groups were PBM and control (no PBM; only orthodontic force) groups. At the first day of start of en-masse maxillary space closure after spring activation (first day, T0) a gallium aluminium arsenic semiconductor diode laser (N-lase, Medsol, Hosur, India) (Figure 2) with wavelength of 810 nm (continuous wave (CW), 200 mW power (0.2W), 0.3cm2 spot size) was used in contact mode for a exposure duration of 8 seconds per spot, at two buccal spots per tooth, two palatal spots to perform PBM treatment immediately and PBM was similarly repeated on the third, seventh, and 14th day. The laser device tip was placed on two buccal points (centre of cervical half and central of apical half) along the mucosa covering the root surface of each of the six maxillary anterior teeth (right canine, right lateral incisors, right central incisors, left canine, left lateral incisors, left central incisors) (Figure 2). Similarly, the laser tip was then used at two palatal (centre of cervical half and central of apical half) points for all teeth mentioned (Figure 2). Thus, all six maxillary anterior teeth were irradiated at four PBM spots, making a total of 24 PBM spots. Total energy delivered per session was 0.2W*four points*8 seconds*six teeth=38.4 joules. Exposure time for the entire PBM treatment at every irradiation appointment was 3.2 minutes (8 seconds*four spots*six teeth=192 seconds). Frequency of PBM was four times in a month, at the first, third, seventh, and 14th days. Figure 3 shows a schematic representation of the PBM applied at 12 points on the buccal aspect and 12 points on the palatal aspect. 

In this study, patients and outcome assessors were blinded to reduce the possibility of performance and detection bias. Patients were not aware if they belonged to the control or laser group. For the purpose of patient/subject blinding, PBM treatment was shown to be performed even in the control group by applying the laser tip on the gingiva along the root surfaces of upper anterior teeth at four points per tooth as mentioned in the PBM protocol, but the device was kept off (sham intervention). The outcome assessors and statistician were also blinded. The operator could not be blinded due to the nature of treatment and the laser intervention.

Ethical statement

Institutional ethics committee clearance letter was received before recruiting patients for the study and informed consent was obtained from all patients.

Follow-up, data collection, and outcome measurements

**Figure 4:**
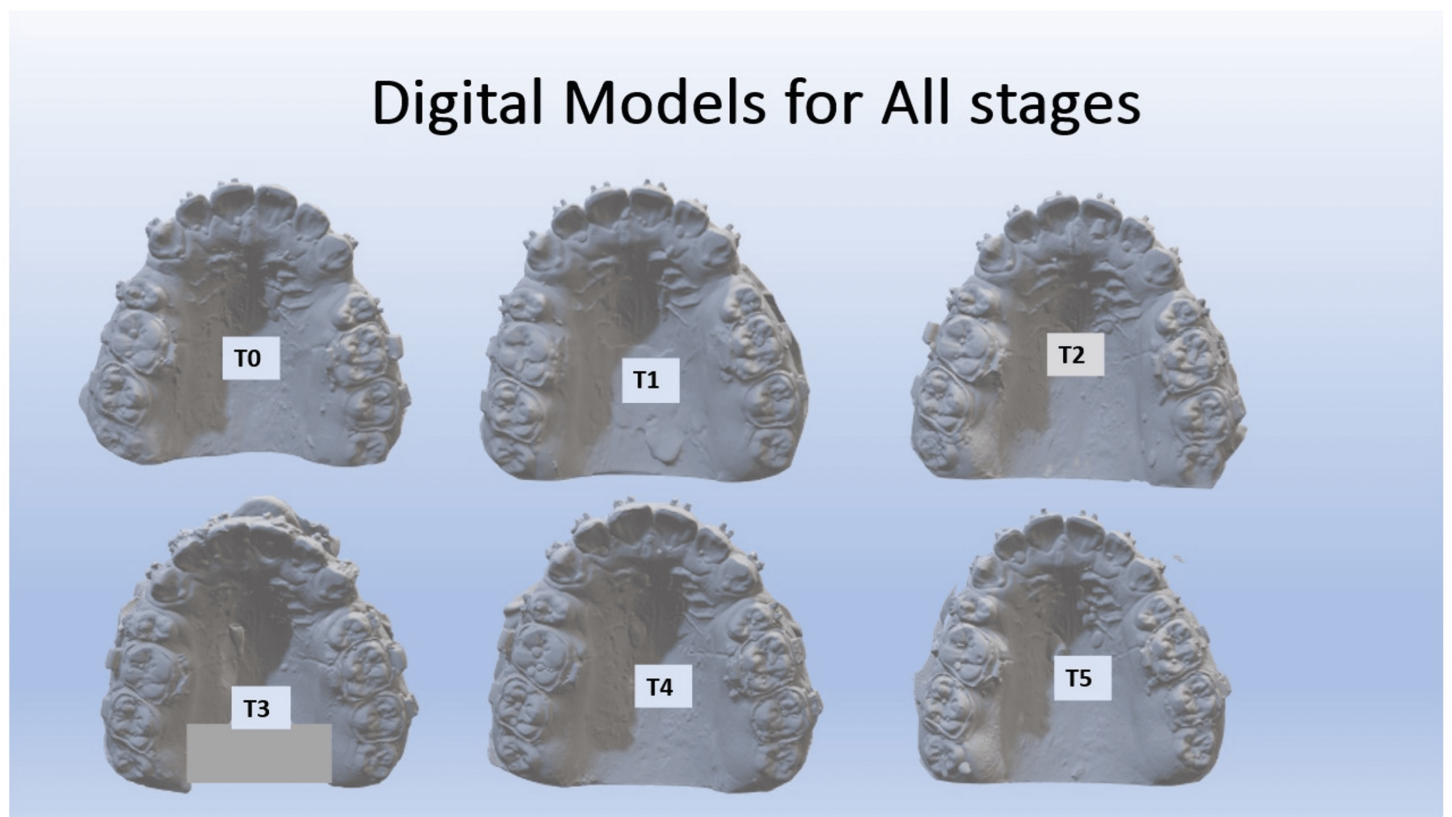
Digital cast images which show monthly progressive space closure distal to the canine (laser group). T0: Start of space closure, T1: End of first month of space closure, T2: End of second month of space closure, T3: End of third month of space closure, T4: End of fourth month of space closure, T5: End of fifth month of space closure

**Figure 5:**
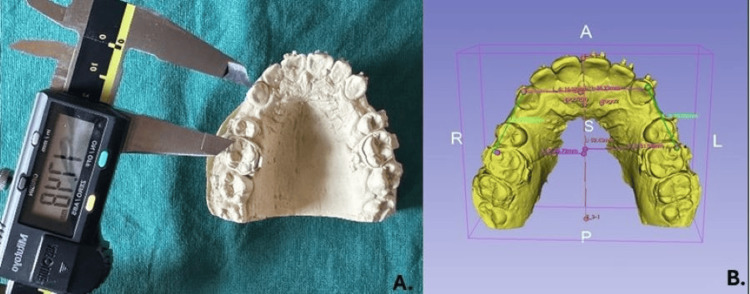
Measurement of tooth movement. A. Measurements done on patient stone models using digital calipers (Method I). B. Measurements done on digital model using 3D Slicer (Version 5.8.1, Slicer Community, USA) (Method II)

A maxillary arch alginate impression was obtained for all patients at the time of first appliance activation, corresponding to the start of maxillary space closure (T0). The impressions were poured immediately, and the resulting casts were scanned to obtain digital models at T0. Monthly follow-up was done. Impressions were repeated at each subsequent monthly appliance activation appointment and monthly casts and digital models were obtained for five successive appliance activation times (T1, T2, T3, T4, T5) (Figure 4). Primary outcome assessment was monthly amount of space closure. The space closure for the first month was measured comparing models between time lines T0-T1. Subsequently, the second month, third month, fourth month, and fifth month space closure was measured between timelines T1-T2, T2-T3, T3-T4, and T4-T5 respectively. On the stone models, the distance between the right maxillary canine cusp tip and mesio-buccal cusp tip of the right molar was measured, using a digital vernier caliper (Method I) (Figure 5). Similarly, measurements were done between the left canine cusp tip and left molar mesio-buccal cusp tip. The space closure measurements were done on the digital models using 3D SLICER version 5.8.1 (Method II). Digital models of all stages in standard tessellation language (STL) format were imported into the software and verified to be correctly scaled in millimetres (sequence of panel tools used: Data → Models → Information). In the presence of any scaling discrepancy, correction was performed using the transform module by adjusting the scale factor. Palatal rugae landmarks, owing to their morphological stability during orthodontic treatment, are considered the gold-standard reference landmarks for study model measurements and superimposition [21]. Calibration was performed using the palatal rugae landmark points and drawing a mid-palatal reference line on each model as described by Anacleto et al. [22]. First, the medial points of the second and third palatine rugae were marked bilaterally and the midpoint between the right and left points was then marked. The median point of the fourth rugae was marked and two points further marked, 5mm posterior and 10mm posterior to the median point of the fourth rugae. All medial points, midline points, and both posterior points were joined to get a median reference line. Tangents were drawn from the median reference line to the canine cusp tip and mesio-buccal cusp tip of the molar on the right and left sides of the maxillary arch. The distance between these tangent points was measured to record the amount of mm space closure monthly (Figure 5). Outcome assessors and data analysts remained blinded to the group assignment of the study subjects. 

Data tabulation and statistical tests

In both the PBM and control groups, all measurements of space closure obtained at monthly intervals for five consecutive months from initiation of en-mass anterior retraction were recorded and tabulated. Mean rate of space closure (in millimetres per month) and corresponding standard deviation (SD) were calculated separately for each monthly interval. Shapiro-Wilk test was used to check data normality. As the data followed normal distribution, for inferential statistical analysis independent samples t-test (unpaired t-test) was applied to compare the mean monthly rate of space closure between Laser and Control group. The threshold adopted for statistical significance was 95% confidence interval (p<0.05). Statistical analyses were performed using IBM SPSS Statistics for Windows, version 25.0 (IBM Corp., Armonk, NY, USA). Statistical analysis followed the intention-to-treat principle (ITT), including all randomized participants in their original groups. If any missing monthly space closure data was reported it was proposed to be addressed using the last observation carried forward (LOCF) method. But in this pilot study, strict adherence to protocol, follow-up appointments and methodology was done. Hence it was not required to do any further sensitivity analysis.

Further, in this study, within-group changes over time were assessed using descriptive statistics and mean rate calculations, and between-group comparisons were made using independent t-tests for each time interval. This approach was appropriate for the limited sample and exploratory nature of the pilot study, focusing on estimating trends and feasibility rather than confirmatory inference. Tests like repeated-measures ANOVA requires several assumptions, including normal distribution, sphericity, and adequate sample size to ensure statistical power. Since this was a pilot study with a small sample size, the data were not suited for parametric repeated-measures analysis.

## Results

Demographics

Ten patients, all females, with an average age of 18.6 years, were recruited and randomly allocated to the control and laser groups. Monthly space closure was assessed in both groups by obtaining alginate impressions and casts and measurements done on the casts. Figure 4 shows the series of monthly scanned digital casts of a patient from the laser group.

Space closure

Overall assessment, that is cumulative space closure over a five-month observation period is included in Tables 2-5. The mean amount of space closure in the maxillary arch on right side between timelines T0 - T1 (First month of space closure) in the control group was 0.533mm (SD 0.045), while in the laser group it was 1.286mm (SD 0.105) with a mean difference of 0.73 (95%CI:0.85-0.61). The difference in the amount of space closure was statistically significant (p<0.05). Further, for all the subsequent monthly time intervals T1 - T2 (Second month), T2 - T3 (Third month), T3 - T4 (Fourth month) and T4 - T5 (Fifth month), a significantly higher amount of space closure was observed in the laser group compared to the control groups in both left and right segments. In Method II, monthly digital models of patients in both the control and laser group were analysed for space closure using the 3D SLICER 5.8.1 software and the results were tabulated. The amount of monthly space closure was observed to be significantly higher for both the right and left maxillary quadrants when assessed by Method I (Table 2, Table 3) and by Method II (Table 4, Table 5) both.

**Table 2:** Results obtained using unpaired t-test for monthly space closure of upper left dental arch with mean values in millimeters, along with standard deviation and statistical significance (p) (Caliper Method I)

Stage/Month	Group	N	Mean (mm)	Std. Deviation	p-value	Mean difference
T_0 _-T_1_	Control	5	0.6243	0.11949	0.002	-0.64812
	Laser	5	1.2724	0.30898		
T_1_-T_2_	Control	5	0.5561	0.04937	0	-0.63164
	Laser	5	1.1878	0.10183		
T_2_-T_3_	Control	5	0.5903	0.07988	0.001	-0.74648
	Laser	5	1.3368	0.3047		
T_3_-T_4_	Control	5	0.514	0.04947	0	-0.63548
	Laser	5	1.1495	0.0695		
T_4_-T_5_	Control	5	0.5358	0.12238	0	-0.67568
	Laser	5	1.2115	0.10748		

**Table 3:** Results obtained using unpaired t-test for monthly space closure of upper right dental arch with mean values in millimeters, along with standard deviation and statistical significance (p) (Caliper Method I)

Stage/Month	Group	N	Mean (mm)	Std. Deviation	p-value	Mean difference
T_0__T_1_	Control	5	0.5333	0.0452	0	-0.73444
	Laser	5	1.2678	0.1051		
T_1__T_2_	Control	5	0.5408	0.0716	0	-0.58428
	Laser	5	1.125	0.0514		
T_2__T3	Control	5	0.5671	0.0299	0	-0.53918
	Laser	5	1.1063	0.1408		
T_3__T_4_	Control	5	0.6227	0.104	0	-0.54374
	Laser	5	1.1665	0.0671		
T_4__T_5_	Control	5	0.7739	0.1163	0.001	-0.35612
	Laser	5	1.13	0.1024		

**Table 4:** Results obtained using unpaired t-test for monthly space closure of upper right dental arch with mean values in millimeters, along with standard deviation and statistical significance (p) (Software Method II)

Stage/Month	Group	N	Mean	Std. Deviation	p-value	Mean difference
T_0 _-T_1_	Control	5	0.5905	0.1075	0	-0.47764
	Laser	5	1.0682	0.15019		
T_1_-T_2_	Control	5	0.5997	0.11696	0	-0.48184
	Laser	5	1.0815	0.09515		
T_2_-T_3_	Control	5	0.5589	0.10838	0	-0.64536
	Laser	5	1.2043	0.16617		
T_3_-T_4_	Control	5	0.5806	0.12685	0	-0.5284
	Laser	5	1.109	0.10505		
T_4_-T_5_	Control	5	0.5456	0.08463	0	-0.47636
	Laser	5	1.022	0.04061		

**Table 5:** Results obtained using unpaired t-test for monthly space closure of upper left dental arch with mean values in millimeters, along with standard deviation and statistical significance (p) (Software Method II)

Stage/Month	Group	N	Mean	Std. Deviation	p-value	Mean difference
T_0 _-T_1_	Control	5	0.5517	0.0761	0.001	-0.56
	Laser	5	1.1117	0.2408		
T_1_-T_2_	Control	5	0.5574	0.0821	0.001	-0.6099
	Laser	5	1.1673	0.2363		
T_2_-T_3_	Control	5	0.5861	0.0844	0	-0.52258
	Laser	5	1.1087	0.0777		
T_3_-T_4_	Control	5	0.4987	0.0891	0	-0.55544
	Laser	5	1.0541	0.0662		
T_4_-T_5_	Control	5	0.6163	0.0622	0	-0.44664
	Laser	5	1.063	0.0695		

The overall assessment, that is, cumulative space closure over five months observation period (T0 to T5), showed that in the upper right quadrant, there was 3.03mm (SD 0.23) of space closure in the control group, while in the laser group it was 5.80mm (SD 0.29) with a mean difference of 2.76 (95%CI:3.14-2.38). The five-month cumulative space closure was significantly higher in the laser group compared to the control. However it is necessary to note the wide CI, which indicates limited precision of the estimate. Though results suggest potential benefit of PBM in accelerating space closure, the findings should be interpreted with caution.

Comparison of monthly space closure measures obtained using methods I and II

Intraclass correlation coefficient (ICC) analysis was performed to assess the reliability and agreement between the two measurement methods - between digital model measurement method and conventional measurement methods and to evaluate the degree of consistency between the measurements obtained using Method I and Method II across all time points. ICC was chosen over the Bland-Altman analysis because the objective was to quantify the degree of reproducibility and internal consistency between the two techniques rather than to evaluate the magnitude of bias.

The ICC (two-way mixed effects model, absolute agreement type) was calculated (Table 6). The conventional threshold for ICC values interpretation is <0.5: poor reliability, 0.5-0.75: moderate reliability, 0.75-0.9: good reliability, >0.9: excellent reliability. The calculated ICC was 0.832, indicating good correlation between the two methods. Average discrepancy on the positive side was 0.153 and on the negative side was -0.108.

**Table 6:** Intraclass correction coefficient (ICC) to compare the correlation between Method I (Caliper Method) and Method II (Software Method) ICC was 0.832, indicating good correlation between the two methods. Average discrepancy on the positive side was 0.153 and -0.108 on the negative side.

Statistic		Positive side	Negative side including zero
Number and percentage		63 (63%)	37 (37%)
Mean		0.153	-0.108
Median		0.112	-0.108
Std. Deviation		0.157	0.070
Minimum		0.002	-0.004
Maximum		0.842	-0.248
Percentiles	25th	0.054	-0.161
	50th	0.112	-0.108
	75th	0.198	-0.047

Safety reporting of PBM

During the five-month observation period of this study, there were no adverse effects like pain, inflammation, or ulceration reported by the patient. However no specific measurement or assessment was done to check adverse effects like root resorption.

## Discussion

The above result clearly indicates that using the specific PBM protocol of 810 nm LLLT (200 mW power for 40 seconds per tooth, 10 seconds at four points along the root surface) irradiation applied for rate of orthodontic tooth movement can be enhanced. In this study we used an irradiation protocol of zero day (appliance activation day), third, seventh, and 14th day irradiation. It is important to understand the rationale behind this protocol and the mechanism of action of LLLT and how it brings about the acceleration in tooth movement. It is also pertinent to know how the effects of photomodulation are different from the other techniques of tooth movement acceleration. During orthodontic treatment, between each appliance activation appointment, tooth movement occurs in different successive phases [23]. From the knowledge of the biology of tooth movement, we know that at the molecular level, each phase of tooth movement is characterised by certain specific cells which are predominant during that phase of movement [24].

When orthodontic force is applied to a tooth, there is immediate microtrauma induced in the supporting periodontal ligament space and adjacent alveolar bone, which causes a zone of hyalinization and necrotic tissue. In the initial phase of one to three days, to remove the hyalinized tissue, it is seen that there is an influx of the osteoclast precursor cells into the periodontal ligament spaces, followed by osteoclast differentiation and maturation and bone resorption and removal of the hyalinized zones, if any, is the major molecular activity. There is a marked increase in the primary messengers and pro-inflammatory cytokines. From the third to seventh day, osteoclastic activity plateaus and goes on at a steadier rate. From the seventh day onwards, the cellular activity changes again with a gradual increase of osteoblastic activity and with more balance seen between osteoclastic activity and osteoblastic activity. From the 14th day onwards, the osteoclastic activity decreases and is overtaken by the osteoblastic activity. Based on these cyclic cellular activity changes, the LLLT irradiation protocol was set on the first, third, seventh, and 14th day for activation of the different cells, which change over the period of tooth movement between two activation appointments of the appliance [25].

Invasive methods of accelerated orthodontics, such as corticotomy, micro-osteoperforations, and piezocision, stimulate trauma-induced osteoclastic activity, resulting in a transient reduction in bone density. This localized bone remodelling is considered the primary mechanism underlying accelerated tooth movement. Various studies have shown that in non-invasive methods like LLLT PBM, both the osteoclastic and osteoblastic activity are stimulated, and this is seen as a change in different salivary and gingival crevicular fluid (GCF) biomarker levels like interleukin-1β, RANK, RANKL, OPG, MMPs, and others [26]. Osteoclastic activity is considered the rate-limiting step for orthodontic tooth movement. Osteoclast maturation, however, needs release of RANKL by the osteoblasts and with use of LLLT using the protocol of four times irradiation (first, third, seventh, and 14th day), it is possible to increase both the osteoclastic and osteoblastic activity [27].

Along with the irradiation frequency protocol, the appropriate dose of LLLT delivered is also important. In this study we used 810 nm LLLT (200 mW power for 40 seconds per tooth, 10 seconds at four points along the root surface) to deliver 8-10 J energy per tooth. The energy delivered was calculated using the formula: Energy = Power out (in W) × Exposure time (in seconds), thus according to the settings used in this study, 0.2 W (Power) × 4 (points-2 buccal and 2 palatal) × 10 seconds = 8 Joules of energy delivered per tooth [28,12]. Further in this study, the space closure was measured using both caliper method and digital models. It has been recently advocated that digital study model analysis techniques are more accurate and predictable for all analysis purposes [29]. Measurements by both methods show that the rate of space closure was found to be significantly higher in the laser group than in the control group.

Though there is a lot of scientific literature on LLLT usage for accelerating tooth movement, the evidence is not coherent, especially in terms of the LLLT protocol to be used. No uniform protocols have been followed in the various previous studies and the studies have been single-tooth movement studies like canine retraction studies [29]. In routine orthodontic treatment for treatment of proclination with premolar extraction, en-masse retraction of the entire anterior segment is preferred as it is a more efficient and simpler method and en-masse retraction compared to canine retraction itself reduces the treatment time. Also, the device used in this study is a simple portable handheld device designed for PBM with wavelengths of 660 and 810 nm at 0.2 W and 0.5 W settings, which makes it convenient for the clinician to use in tissue contact mode. With the use of protective eyewear for both the operator and patients, no adverse effects were reported [30].

Strength and limitations

This pilot study addressed a clinically relevant research question with a clearly defined and measurable primary endpoint. Strengths include a randomized, parallel-group design with a control and sham intervention, standardized treatment protocols and follow-up visits, and blinded outcome assessment, all of which enhance internal validity and reduce expectation and measurement bias. Monthly evaluation of tooth movement allowed objective and reproducible outcome assessment, while the pilot design facilitated assessment of feasibility and outcome variability for future trials.

Limitations include the small sample size, which limits statistical power, and the single-centre design with short follow-up, restricting external validity and long-term safety assessment. Inclusion of only female participants limits generalisability, and the parallel-arm design permits interindividual biological variability to act as a confounder. Biological markers and radiographic adverse effects, including root resorption, were not evaluated. Thus, the findings are hypothesis-generating and require confirmation in larger, pre-registered studies.

## Conclusions

The results of this pilot study show that with the PBM device N-lase and PBM protocol to deliver 6.4 J energy per tooth on the first, third, seventh, and 14th days of the monthly appliance activation cycles, the orthodontic tooth movement can be accelerated during space closure, and this has a significant clinical implication that the overall treatment time can be reduced by almost 40%. Considering the small size, wide CI in results, and other limitations of this randomized pilot study, a preliminary hypothesis can be derived that, when PBM was added to standard orthodontic force with the N-lase device, a higher monthly rate of extraction space closure was seen. With this, the treatment time can be reduced, and this helps in providing better quality care for the patients.
